# COVID-19 and andrology: Recommendations of the French-speaking society of andrology (Société d’Andrologie de langue Française SALF)

**DOI:** 10.1186/s12610-020-00106-4

**Published:** 2020-07-13

**Authors:** S. Hamdi, M. Bendayan, E. Huyghe, J.-C. Soufir, E. Amar, R. El Osta, I. Plotton, C. Delalande, J. Perrin, C. Leroy, A. Bouker, H. Pons, H. Lejeune, G. Robin, F. Boitrelle

**Affiliations:** 1 Société d’Andrologie de langue Française (SALF), http://www.salf.fr; 2 Université de Toulouse, UPS, Groupe de Recherche en Fertilité Humaine (EA 3694, Human Fertility Research Group), 330 avenue de Grande Bretagne, 31059 Toulouse, France; 3 Unité d’Andrologie, Groupe d’activité de médecine de la reproduction, Hôpital Paule de Viguier, CHU Toulouse, 330 avenue de Grande Bretagne, 31059 Toulouse, France; 4 Service de biologie de la reproduction-Préservation de la fertilité- Andrologie, Hôpital de Poissy Saint Germain en Laye, 78300 Poissy, France; 5 Université Paris-Saclay, UVSQ, INRAE, BREED, 78350 Jouy-en-Josas, France; 6 École Nationale Vétérinaire d’Alfort, BREED, 94700 Maisons-Alfort, France; 7 Service d’AMP et Préservation de la Fertilité, Hôpital Jeanne de Flandre, CHU de Lille, 59037 Lille cedex, France; 8 Service d’Andrologie, Hôpital Claude Huriez, CHU de Lille, 59037 Lille cedex, France; 9 Lille University, EA4308 “Gametogenesis and gamete quality”, 59120 Loos, France

**Keywords:** Covid-19, SARS-CoV-2, Testis, Semen, Orchitis, Fever, Testosterone, Andrological care, Male, Assisted reproductive technologies, Covid-19, SARS-CoV-2, Testicule3, >Orchite, Sperme, Fièvre, Testostérone, Suivi andrologique, Homme, Assistance médicale à la procréation

## Abstract

SARS-CoV-2 (severe acute respiratory syndrome coronavirus 2) metamorphosed our medical practice. In early June 2020, more than 6,400,000 COVID-19 (coronavirus-19 disease) cases were diagnosed across the world and more than 380,000 deaths were linked to COVID-19. Many medical symptoms of COVID-19 were reported. We will focus, here, on potential impacts of COVID-19 on men’s andrological health. Our society (French-speaking society of andrology, SALF) also emitted some recommendations in the andrological management of men infected by SARS-CoV-2. First, considering the fever and the potential presence of SARS-CoV2 in semen, SALF recommends waiting for 3 months (duration of one spermatogenesis cycle and epididymal transit) before re-starting ART in the case of men diagnosed COVID-19 positive. Whatever the nature of testosterone and COVID-19 relationships, we recommend an andrological examination, sperm parameters, and hormonal evaluation at the time of the COVID-19 is diagnosed, and several months later. Furthermore, we are concerned by the potential morbid-mortality of the COVID-19, which mainly affects men. This “andrological bias”, if proven, must be reduced by specific andrological diagnosis, therapeutic and prophylactic measures. Research in this direction must be substantiated and financially supported over the next few months (years).

## Resume

Le SRAS-CoV-2 (nouveau coronavirus ou coronavirus numéro 2 responsable du syndrome respiratoire aigu sévère) a métamorphosé notre pratique médicale. Début juin 2020, plus de 6,400,000 cas de COVID-19 (maladie à coronavirus 2019) ont été diagnostiqués dans le monde et plus de 380,000 décès ont été reliés à cette maladie. De nombreux symptômes médicaux de cette infection virale ont été signalés. Nous nous concentrerons, ici, sur les impacts potentiels de COVID-19 sur la santé andrologique des hommes. Notre société (Société d’andrologie de langue Française, SALF) émet ici quelques recommandations dans la prise en charge andrologique des hommes infectés par le SRAS-CoV-2. Tout d’abord, compte tenu de la fièvre et de la présence potentielle du SRAS-CoV2 dans le sperme, la SALF recommande d’attendre 3 mois (durée d’un cycle de spermatogenèse et transit épididymaire) avant de recommencer les techniques d’assistance médicale à la procréation pour les hommes diagnostiqués COVID-19 positifs. Quelle que soit la nature des relations entre la testostérone et l’infection à SARS-CoV-2, nous recommandons un examen andrologique, un examen des paramètres du sperme et une évaluation hormonale au moment du diagnostic de l’infection, ainsi qu’à distance (3–6 mois plus tard). De plus, nous sommes préoccupés par la morbidité et la mortalité potentielles de l’infection COVID-19, qui touche principalement les hommes. Ce “biais andrologique”, s’il est. prouvé, doit être réduit par un diagnostic andrologique spécifique et des mesures thérapeutiques et prophylactiques. La recherche dans ce sens doit être étayée et soutenue financièrement au cours des prochains mois (années).

## Background

In just a few months, COVID-19 spread in more than 200 countries, and to date, more than 6,400,000 patients were infected and more than 380,000 deaths were reported worldwide [1]. This viral pandemic metamorphosed our medical practice and compelled academic health systems and other clinical settings to move forwards [2]. According to the Global Health 5050, an independent organization that promotes gender equality in healthcare and gathers sex-disaggregated data about COVID-19, there is not a clear pattern in terms of whether women or men are more likely to be diagnosed with COVID-19. However, in most countries where data were available, the mortality rate is higher among men [3, 4]. Early case series reported indeed that most of the infected patients with SARS-CoV2 were men [5, 6] and further studies confirmed that infected men presented more severe diseases (i.e. intensive care units’ admission) and a higher mortality than women [7–9] despite the same initial prevalence [10].

Thus, a COVID-19 gender-gap in health is emerging. As a medical and scientific society dedicated to andrology for nearly 40 years (French-speaking society of andrology, SALF), we would like to contribute to the global effort to explain and try to reduce this gap. Here, we would like to highlight some specific impacts of the SARS-CoV-2 on male health and then present our recent recommendations on the andrological management of men infected by SARS-CoV-2 during assisted reproductive technologies (ART) or andrological evaluation.

## Main text

### Impacts of COVID-19 infection on testis and semen

Firstly, fever was defined as a major symptom of COVID-19 that complicated more than 80% of cases [11]. Fever alone could impact spermatogenesis and thus alter sperm parameters such as sperm concentration. Fever is also known to negatively impact sperm motility and to alter sperm DNA integrity [12]. The delay to sperm parameters recovery after COVID-19 fever is still unclear. Is this 72–90 days, as previously described [12, 13], more? or less? In our opinion, according to the current state of knowledge, waiting for 3 months could probably allow a baseline-return of sperm parameters after fever in COVID-19.

The second question to answer is: could SARS-CoV-2 infect the testis? and, if so, what could it cause? The viral entry into target cells depends on the SARS-CoV-2 spike (S) protein, allowing the fusion of viral and cellular membranes [14, 15]. The SARS-CoV-2 virus employs angiotensin-converting enzyme 2 (ACE2) receptors to enter the human cells and the transmembrane serine protease (TMPRSS2) allows S protein priming [14, 16]. A recent in silico analysis [17] suggested that a high affinity of SARS-CoV-2 S protein in the interaction with the human ACE2 receptor could explain the pandemic status of COVID-19.

The male reproductive system expresses both ACE2 receptor and TMPRSS2 receptors [14, 18–20]. Several studies detected indeed high ACE2 receptor expression level in testicular cells, mainly in spermatogonia, Leydig cells, and Sertoli cells; these later cells were described as having the highest expression level [19, 21]. Interestingly, the younger the patient, the higher ACE2 receptor expression; suggesting a higher risk of testicular damage by COVID-19 in young patients than in older patients [19].

Hence, SARS-CoV-2 could potentially act on testicular cells. Several cases of orchitis have been indeed described in the case of another SARS-CoV, although the virus itself was not isolated in the testicular tissue. There was on these patients, an extensive IgG precipitation in testicular tissue causing germ cell destruction and testicular leukocyte infiltration [22], suggesting a possible immune response as the cause of the testicular damage. Given the degree of homology between coronaviruses (SARS-CoV-2 and/or its receptor sharing high homology with other SARS viruses and/or receptors [23]), SARS-CoV-2 could also lead to orchitis, even if this was not yet described. Hence, it is not unreasonable to think that the major inflammation associated to SARS-CoV-2 might transiently affect blood-testis barrier integrity and may affect spermatogenesis. As a result, we advise to investigate urogenital and andrological manifestations in COVID-19 infected men, who could complain of scrotal or urogenital discomfort or pain.

Finally, the burning question is now: could we find virus in the semen of infected COVID-19 patients? Does the virus pass the blood-testis barrier or could it be excreted in prostatic or seminal vesicle fluid and be found in the seminal fluid? Is SARS-CoV-2 sexually transmittable? Answering these questions will certainly take time.

Four studies did not identify the virus in the semen of a total of 68 COVID-19 infected patients [24–27]. One Italian patient provided a semen sample, 15 days from the onset of the COVID-19. No virus was detectable in his ejaculate [24]. For 12 other patients, no virus was detected within semen in their recovery phase, as well as in testicular samples from one patient who died of COVID-19 during the acute phase [25]. In the case of 34 Chinese patients recovering from COVID-19, no virus was found in semen samples [26]. It should be noted that most of these patients have presented mild forms of COVID-19 and that SARS-CoV-2 semen analysis was done about 1 month after COVID-19 infection (median 31 days). Using single-cell RNA (ribonucleic acid) sequencing (scRNA-Seq) cellular data showing that only 4 out of 6490 (< 0.1%) testicular cells contained RNA for both ACE2 receptor and TMPRSS2 in a previous study, the authors concluded that it appeared unlikely that SARS-Cov-2 could enter into any cells in the testis as it had previously been hypothesized. The same results were reported by Stanley et al. [28] who did not observe a large number of testicular cells expressing both ACE2 receptor and TMPRSS2; it should however be noted that Sertoli cells were under-represented among testicular cells analyzed in this latter study. Finally, for 20 men (18 COVID-19 recovered patients with semen samples collected between 1 to 8 weeks 8 after an absence of symptoms and 2 patients with COVID-19 infection at the acute stage), no SARS-CoV-2 RNA was detected in the semen. Patients with moderate symptoms showed an impairment of semen parameters quality (mainly alteration of semen total sperm count and motility) but no semen analysis was available before COVID-19 [27].

If these data are reassuring, a recent manuscript mentioned virus in semen [29]. The virus was found in the semen of 6 out of 38 COVID-19 patients. Four patients were at the acute stage of the disease and 2 were considered as clinically recovered (12 and 16 days after the onset of symptoms). Since ACE2 and TMPRSS2 could be expressed in prostate epithelial cells [30], the presence of SARS-Cov-2 in seminal fluid could result from prostate infection. Whatever its origin, the virus could potentially be present in semen. In which form? Complete? or not? Does this presence mean infectious potential and sexually transmittable capacity? Maybe not but these questions could be asked and remain unanswered. It should also be noted that the diagnostic accuracy of many of the commercial qRT-PCR (quantitative reverse transcription polymerase chain reaction) kits available for detection of SARS-CoV-2 may be lower than optimal (i.e., sensitivity and specificity < 100%). Therefore, false positive and also false negative results could be obtained. So, to the question “could there be virus in the semen”, we cannot answer with certainty.

### Impacts of COVID-19 on infertile men’s management during assisted reproductive technologies

What are the effects of the virus on sexual and reproductive health? Social distancing measures could potentially impact the patients’ sexual quality of life. Concerning reproductive health, assisted reproductive technologies (ART) have been drastically reduced since the pandemic COVID-19 beginning as in early March 2020, nothing was known about SARS-CoV-2 impacts on pregnancy and gametes. In Europe, several countries were particularly affected by COVID-19. Hence, in March, 2020, several scientific societies recommended stopping non-urgent ART. In France, the Biomedicine Agency (ABM) imposed the suspension of ART, except for oncological sperm cryopreservation. Delaying care of infertile patients could affect their parenthood capacity as suggested by Esteves et al. [31]. In this context, French-speaking Andrology society (SALF) and other national scientific societies participated in the elaboration of recommendations at the time of restarting ART in France. These recommendations were published on May, the 25th, on the ABM website and will be updated as necessary.

What is clear for ART management of patients? Viable virus could be detected in aerosols for up to 3 h, and on surfaces for days after application [32]. Given this, it is recommended: to be extra careful with the disinfection of sperm retrieval rooms; to pay attention to renew the air between two patients; and to insist on the interest of wearing a mask during retrieval to avoid the contamination of the semen.

What remains unclear for ART management of patients? Concerning men and the potential presence of SARS-CoV-2 in semen, SALF recommends waiting for about 3 months before ART is carried out on men diagnosed with COVID-19. This delay was assessed considering (i) the potential effect of fever on sperm parameters as previously described and the (ii) potential presence of SARS-CoV-2 in the semen of COVID-19 recovered patients. To assess the impact of fever on semen parameters, semen parameters could be assessed at different times to determine the time of baseline-return of these parameters. These controls could then potentially reduce the time of ART-no use of semen below 3 months. Otherwise, as it has been described for other viruses, sperm preparation during ART could potentially decrease the SARS-CoV-2 presence (if it exists) on sperm used for In vitro fertilization or intrauterine insemination. Nevertheless, no study has yet assessed this hypothesis. Finally, even if SARS-CoV-2 potential to catch on spermatozoa is not expected, it was not assessed yet. As a result, a 3 months-ART reporting in COVID-19 patients could appear (too) long but given that literature data are rare, it is a precautionary principle that SALF assumes until literature data increases.

### Impacts of COVID-19 on male hormonal profile: a complex interaction

The difference in the rate of severe COVID-19 cases between men and women was first linked to comorbidities and behavioral risk factors (smoking, fewer hands washing …) but two preliminary observations rapidly drew attention to a link between androgens and COVID-19 pathogenesis.

First, among Spanish males hospitalized for bilateral SARS-CoV-2 pneumonia, a high proportion was diagnosed with a pattern of androgenetic alopecia [33]. Secondly, an Italian population-based study reported that, comparing the total number of SARS-CoV-2 positive cases, prostate cancer patients receiving androgen-deprivation therapy had a significantly lower risk of infection compared to patients who did not receive it [34]. Thus, testosterone was seen as a promotor of severe COVID-19 infection and proposals to use androgen suppression to reduce COVID-19 vulnerability emerged [35, 36]. Published and not yet published molecular data about the cell-machinery that permits SARS-CoV-2 entry, mainly ACE2 and TMPRSS2, fueled also this hypothesis of the highest male-related susceptibility: ACE2 gene is located on the X chromosome and TMPRSS2 expression could be androgen-dependent in several tissues, including lungs [37, 38].

On the other hand, two studies explored the hormonal status of male patients during COVID-19. One non-peer reviewed study examined the impact of SARS-CoV-2 on male sex hormones in 81 COVID-19 patients compared to 100 age-matched healthy men [39]. Serum *luteinizing hormone* (LH) levels were significantly higher in the COVID-19 group, although all LH-levels remained in normal ranges. More interestingly, another study determined LH, total testosterone, sex Hormone Binding Globulin (SHBG), and calculated free testosterone for 31 patients affected by SARS-CoV-2 pneumonia admitted in a respiratory intensive care unit [40]. Significantly higher LH levels (above normal ranges in average), significantly lower total and free testosterone levels (under normal ranges in average) were found in the group of patients with a higher risk of clinical deterioration (including death), compared to the group of patients with a more stable condition. No difference was observed concerning SHBG levels. Thus, another question emerges: is hypogonadism a cause for or a consequence of male patients’ COVID-19 worse symptoms? It is well known that low levels of testosterone, like those of aged males with comorbidities, are linked to defective lung functions and increased pro-inflammatory cytokines; two conditions that may favor severe COVID-19 infection [35]. In another hand, given that Leydig cells express ACE2 and TMPRSS2 [19, 21, 41], it may be possible that testosterone secretion is impaired by the SARS-CoV-2.

This complex interaction between testosterone and COVID-19 deserves strong research efforts on both clinical and biological fronts as suggested by other andrologists [42]. For instance, studies that longitudinally assess and compare testosterone levels in COVID-19 positive male patients with those of age- and comorbidities-matched control groups are still lacking. Whatever the nature of testosterone and COVID-19 relationships, we recommend an andrological examination, sperm parameters, and hormonal evaluation at the time of COVID-19 diagnosis in men, and several months later. Moreover, we need more knowledge to understand how testosterone treatment and/or anti-androgens therapy could be used for prophylactic and therapeutic purposes [43].

## Conclusions

The SARS-CoV-2 epidemic has disrupted health care systems around the world. ART and andrological practitioners were forced to stop their activities, leaving many patients and couples stranded. Recovery is complicated by strict health measures and limited consolidated data on how the virus is transmitted. Nevertheless, several scientific societies, including SALF, have issued recommendations to allow couples to re-start ART treatments. Considering the fever and the potential presence of SARS-CoV2 in semen, SALF recommends waiting for 3 months (duration of one spermatogenesis cycle and epididymal transit) before re-starting ART in the case of men diagnosed COVID-19 positive. Whatever the nature of testosterone and COVID-19 relationships, we recommend an andrological examination, sperm parameters, and hormonal evaluation at the time of the COVID-19 is diagnosed, and several months later. Furthermore, we are concerned by the potential morbid-mortality of the COVID-19, which mainly affects men. This “andrological bias”, if proven, must be reduced by specific andrological diagnosis, therapeutic and prophylactic measures. Research in this direction must be substantiated and financially supported over the next few months (years).

## Data Availability

Not applicable.

## Abbreviations

ABM: Agence de la Biomédecine (Biomedicine Agency)
ART: Assisted reproductive technologies
ACE2: Angiotensin-converting enzyme 2
COVID-19: Coronavirus disease 2019
LH: Luteinizing hormone
qRT-PCR: Quantitative reverse transcription polymerase chain reaction
RNA: Ribonucleic acid
SALF: French speaking society of Andrology
SARS-CoV-2: Severe acute respiratory syndrome coronavirus 2
SHBG: Sex Hormone Binding Globulin
scRNA-Seq: Single-cell RNA (ribonucleic acid) sequencing
TMPRSS2: Transmembrane protease serine 2
